# Frequency of osteoradionecrosis of the lower jaw after radiotherapy of oral cancer patients correlated with dosimetric parameters and other risk factors

**DOI:** 10.1186/s13005-022-00311-8

**Published:** 2022-02-26

**Authors:** Kristin Lang, Thomas Held, Eva Meixner, Eric Tonndorf-Martini, Oliver Ristow, Julius Moratin, Nina Bougatf, Christian Freudlsperger, Jürgen Debus, Sebastian Adeberg

**Affiliations:** 1 Department of Radiation Oncology, University Hospital of Heidelberg, Im Neuenheimer Feld 400, 69120 Heidelberg, Germany; 2 Heidelberg Institute of Radiation Oncology (HIRO), 69120 Heidelberg, Germany; 3 Department of Oral and Maxillofacial Surgery, University Hospital Heidelberg, Im Neuenheimer Feld 400, 69120 Heidelberg, Germany; 4 Heidelberg Ion Therapy Center (HIT), Im Neuenheimer Feld 450, 69120 Heidelberg, Germany; 5 Clinical Cooperation Unit Radiation Oncology, German Cancer Research Center (DKFZ), Im Neuenheimer Feld 280, 69120 Heidelberg, Germany

**Keywords:** Desolate dental status, Osteoradionecrosis, Radiotherapy, Mandibular bone

## Abstract

**Objectives:**

Osteoradionecrosis (ORN) of the lower jaw is a serious late complication after radiotherapy in patients with oral cavity cancer. The aim of this study is to generate more insight into which patient- and treatment-related factors are associated with the development of ORN in oral cavity cancer patients undergoing postoperative radiotherapy.

**Material and methods:**

Retrospective evaluation and comparison of 44 patients with ORN (event group 1) matched according to 45 patients without ORN (control group 2) who received postoperative radiotherapy of oral cavity squamous cell carcinoma at our institution between 2012 and 2020. Dosimetric factors that favor the occurrence of ORN should be detected. The cumulative occurrence rate of ORN was calculated according to the Kaplan–Meier method and analyzed by Cox regression and log-rank test.

**Results:**

The median time to develop ORN was 18 months (3–93 months) after radiotherapy. Dental status before radiotherapy (RT) treatment (HR 4.5; 1.8–11.5) and dosimetric parameters including Dmean > 45 Gy (HR 2.4; 1.0–5.7), Dmax > 60 Gy (HR 1.3; 1.1–2.8) and planning target volume (PTV) proportion > 40% intersection with the lower jaw (HR 1.1; 1.0–1.1) were significantly associated with ORN.

**Conclusion:**

The results of this retrospective study reveal that oral cavity cancer patients who underwent pre-RT dental surgery as well as dosimetric parameters using Dmax > 60 Gy, higher mean doses > 45 Gy and more than 40% PTV intersection with the lower jaw bone are independent risk factors for ORN. These findings can assist in the management of patients undergoing RT for head and neck cancer regarding ORN prevention.

**Clinical relevance:**

Poor oral hygiene and desolate dental status as well as high radiation doses to the mandibular bone significantly increase the risk of developing osteoradionecrosis. Before irradiating a patient with oral cavity cancer, an appointment with the dentist should be made and teeth sanitized if necessary. Likewise, maximum radiation doses to the lower jaw should be minimized.

## Introduction

Oral cancer is sensitive to radiation and is standard treatment either in definitive intention or in the case of pathologic risk factors (positive resection margin, positive lymph nodes, locally advanced disease) as postoperative intention. Osteoradionecrosis (ORN) is a feared late complication of radiotherapy (RT) of oral cancer patients which affects mostly the lower jaw between 2 and 22% [1–3]. The incidence of ORN is about 4–8% [4–6]. ORN is defined as exposed irradiated bone that fails to heal over a period of 3 months without any evidence of persisting or recurrent tumor [7, 8]. Radiological evidence of bone necrosis within the target volume is also important for diagnosis and classification [9]. Analysis of epidemiological studies of ORN does not offer accurate data about incidence and prevalence of ORN in the jaws because of inconsistencies in the length of follow-up between studies and limited data from prospective studies. Different treatment-, tumor- and patient-related risk factors of ORN have been reported: size and site of the tumor, age, total RT dose, treatment technique (3D-conformal therapy (3D-CRT) vs. intensity-modulated radiotherapy (IMRT)), dose volume histogram (DVH) parameters, dose per fraction, injury or dental extractions, alcohol and tobacco abuse, tumor size or stage, association of the tumor with bone and dental hygiene [10–14]. The international standard radiation technique for patients treated for oral cancer is IMRT, primarily aimed at reducing dosage at the major salivary glands in the oral cavity. Therefore, it is not uncommon to achieve high-dose gradients across the lower jaw bone [15]. All patients treated with RT in the oral cavity underwent pre-RT dental evaluation and pre-RT care if necessary according to current uniform policies. The aim of this study is to generate more insight into which patient- and treatment-related factors are associated with the development of ORN in oral cavity cancer patients undergoing postoperative RT and to predict which patients are at higher risk of developing ORN.

## Material and methods

Data from 89 patients who received postoperative RT to the oral cavity between 2012 and 2020 at the University Hospital of Heidelberg were reviewed for this retrospective analysis. Patient data were homogenized by selecting the same tumor stage and only patients with oral cancer. To ensure comparability, and in particular to be able to detect risk factors, patients were divided into two equal groups: those who developed ORN (44 patients, group 1) and those who did not develop ORN (45 patients, group 2). We collected basic patient and treatment data from the National Tumor Center Heidelberg Cancer Registry and imported them into our HIRO Research Database for this study [16]. Inclusion criteria were as follows: histologically squamous cell carcinoma, follow-up duration of at least 3 months after completion of RT, no interruptions during RT, regular follow-up examinations including computed tomography (CT) examinations every 3 months in the first 2 years after RT as standard clinical practice for all head and neck cancer patients, every 6 months in years 3 and 4 after RT and once a year in years 5 and 6, as well as regular head and neck examinations at the Department of Oral and Maxillofacial Surgery and the availability of sufficient RT treatment plan data to evaluate the dose to the lower jaw bone. Exclusion criteria were patients with follow-up of less than 3 months as well as patients with other histology and patients with cancer outside the oral cavity.

### Treatment features

Before treatment, patients generally underwent pre-RT dental evaluation and pre-RT dental treatment (from attempts to preserve teeth to extraction of teeth not worth preserving), as deemed appropriate by the oral and maxillofacial surgeons, based on risk assessment. The pre-RT dental treatment because of poor dental status was done 2 weeks before the start of radiation. A desolate dental status was defined in our sample as follows: periodontal disease, carious changes in more than 5 teeth, attacked gums or exposed tooth necks. In our sample, patients with caries deep into tooth pulpa were involved. There was no special grading system used for degree of carious changes. For periodontal status the oral and maxillofacial surgeons used periodontal risk assessment (RPA), which includes number of teeth and implants, number of missing teeth, percentage of alveolar bone loss and number of periodontal pockets with probing depths > 5 mm.

Patients were treated either with IMRT or 3D-CRT. CT simulation was performed in patients immobilized using a thermoplastic mask. Target volume definition was performed in accordance with current guidelines [17, 18]. Our target definition was based on preoperative and postoperative CT and magnetic resonance imaging (MRI) scans and included the primary tumor region initial gross tumor volume (iGTV) according to the International Commission on Radiation Units and Measurements definition [19]. The planning target volume (PTV) was created by expansion of the clinical target volume (CTV) with a 3–5 mm uniform margin. The lymphatic CTV encompasses the pathologic lymph nodes (pN+) as well as any adjacent regions at risk of tumor spread. The lower jaw was contoured for each plan, and DVHs were retrospectively reviewed as RT dosimetric parameters. The maximum dose to the lower jaw (Dmax), mean dose to the lower jaw (Dmean) and PTV correlation to lower jaw were reviewed. The main treatment features are listed in Table 1.

**Table 1:** Characteristics of 44 patients with osteoradionecrosis (group 1) and 45 patients in control group (group 2) after postoperative radiotherapy

	Group 1 (%)	Group 2 (%)	*p*-value
Median age at RT in years (range)	70.5 (39–94)	71 (42–88)	0.886
Gender			
Male	35 (79.5)	37 (82.2)	0.124
Female	9 (20.5)	8 (17.8)	
T-stage			
1–2	28 (63.6)	32 (71.8)	0.086
3–4	21 (47.7)	13 (28.9)	
Desolate dental status (caries, periodontal disease)	36 (81.8)	17 (37.7)	0.228
Need for pre-RT dental treatment	31 (70.5)	12 (26.7)	0.001
Tooth protection during RT	41 (93.2)	40 (88.9)	0.303
Systemic therapy			
CHT	22 (50.0)	28 (62.2)	0.475
IT	8 (18.2)	1 (2.2)	
none	14 (31.8)	16 (35.5)	
Smoking history			
yes	29 (65.9)	19 (42.2)	0.453
no	15 (34.1)	26 (57.7)	
RT technique			
3D-CRT	7 (15.9)	2 (4.4)	0.519
IMRT	37 (84.1)	43 (95.6)	
D_mean_ to mandible			
< 45 Gy	25 (56.8)	41 (91.1)	0.023
> 45 Gy	19 (43.2)	4 (8.9)	
D_max_ of mandible			
< 50 Gy	2 (4.5)	10 (22.2)	0.033
51–60 Gy	22 (50.0)	19 (42.2)	
> 60 Gy	20 (45.5)	16 (35.5)	
PTV dimension (ccm) (range)	804 (68–4838)	796 (170–1389)	0.028

### Definition of ORN

Various definitions of ORN have been proposed, but no current accepted standard of classification or grading exists. The most prevalent definition is exposed bone after RT that fails to heal over a period of 6 months without evidence of persisting or recurrent tumor and required drug therapy or surgery with radical mandibulectomy [20].

### Treatment toxicity

Toxicity was evaluated in this retrospective analysis with focus on ORN and was described according to the Common Terminology Criteria for Adverse Events (CTCAE) criteria (version 5.0, U.S. Department of Health and Human Services, Washington, DC, USA): grade 1: asymptomatic; clinical or diagnostic observations only; intervention not indicated; grade 2: symptomatic; medical intervention indicated (e.g., topical agents); limitations in the performance of instrumental activities of daily living (ADLs); grade 3: severe symptoms; limitations in the performance of self-care ADLs; elective operative intervention indicated; grade 4: life-threatening consequences; urgent intervention indicated; and grade 5: death [21]. This retrospective study included all patients who had medical intervention for ORN.

### Statistical analysis

Univariate and multivariate Cox proportional hazard models were used to assess patient- and treatment-related factors associated with the development of ORN. Factors with statistical significance in the univariate analysis were included in the multivariate analysis. The cumulative incidence was estimated using the Kaplan–Meier method, and the log-rank test was used to compare cumulative incidence curves. The follow-up time was calculated from the last date of RT until the most recent follow-up visit at our institution or the date of death. The time to ORN development was calculated from the last date of RT until the date of ORN occurrence. The Mann–Whitney U test was used to compare patients in terms of the presence of ORN, RT technique and DVH parameters. Cut-off points for DVH parameters were selected. All statistical analyses were performed using IBM SPSS software version 24. *P*-values of 0.05 or less were considered statistically significant.

## Results

**Fig. 1:**
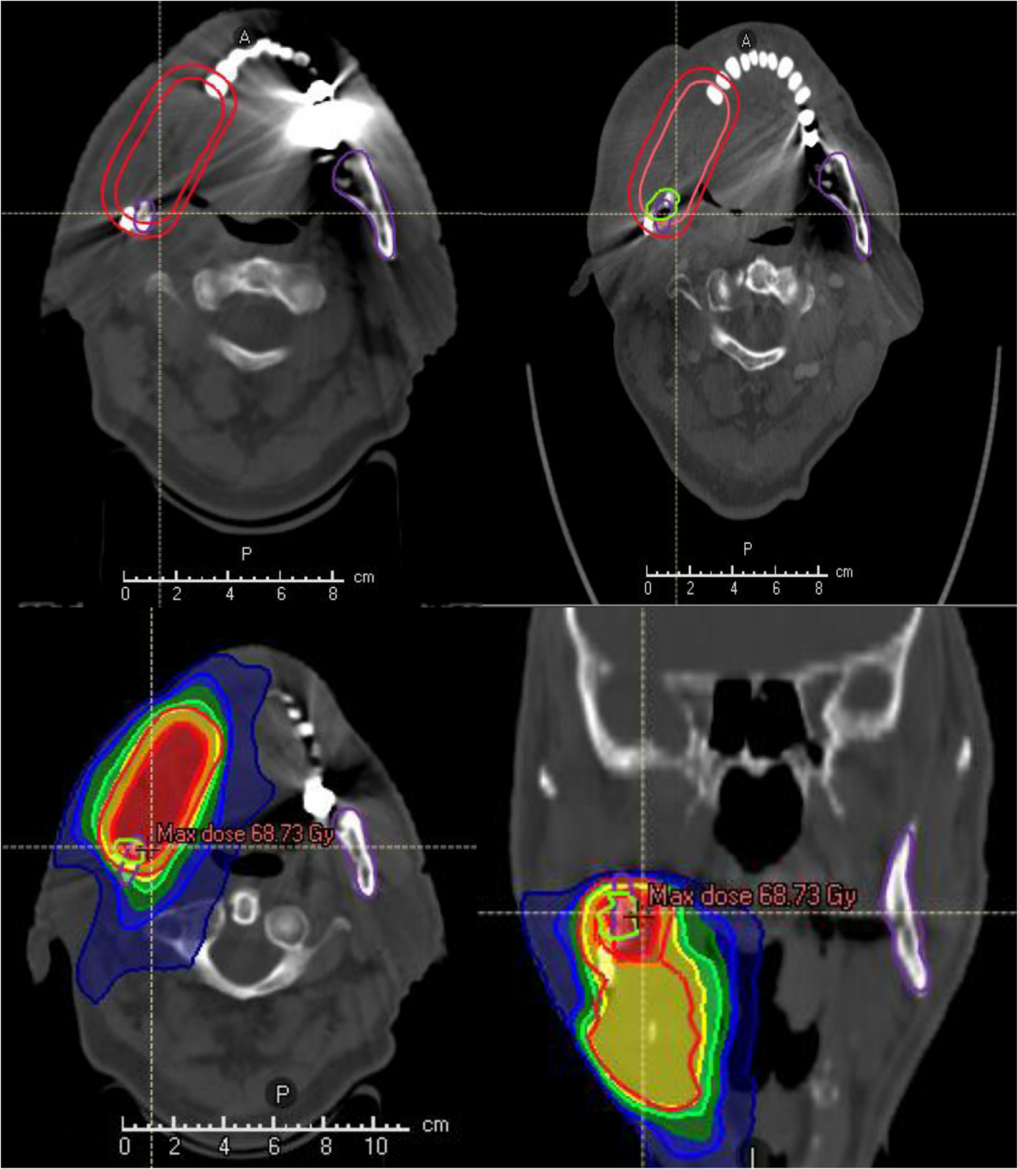
The images a–d show the planned CT in one patient with oral cavity cancer undergoing postoperative radiotherapy, including PTV that was planned to receive a total dose of 66.0 Gy (single dose 2.0 Gy) in 33 fractions. To the top right image b shows the ORN of the lower jaw (colored in green) in the high-dose region with maximum dose 68.7 Gy. Therefore, we matched the follow-up CT with the planned CT and dose distribution, and then volumetric and dosimetric evaluations were performed

Results are shown according to presence of ORN (group 1: with ORN; group 2: without ORN). Patient and treatment characteristics are summarized in Table 1. In both groups there were five patients with desolate dental status included who declined pre-RT dental treatment as preferred. An example for development of osteoradionecrosis is shown in Fig. 1.

The follow-up time for the whole cohort was 28 months, and the median time to develop ORN was 18 months (3–93 months). In total, 78% of 44 ORN sites were located in the body of the jaw, and 22% were located in the angle or ramus. CT scans were evaluated for all ORN sites, and cortical erosion was observed in 73% and loss of spongiosa trabeculation in 27%. Of the 44 patients with ORN, 20 (45.5%) had CTCAE grade 2, 17 (38.6%) had grade 3 and 7 (15.9%) had grade 4. All patients received initial anti-infective therapy with chlorhexidine rinsing (0.2% chlorhexidine solution; GlaxoSmithKline Consumer Healthcare GmbH & Co. KG) and antibiotic treatment, followed by surgical intervention in the infection-free interval. In the time between completion of RT and detection of ORN, no patient had any other dental intervention. For the whole cohort there was no correlation between dental intervention before RT and occurrence of ORN detected.

### Group 1

The median patient age at the time of RT was 71 years (range 39–94 years); 35 patients (79.5%) were male and 9 (20.5%) were female. Pre-RT dental treatment was necessary in 31 patients (70.5%), with half of them having 3 or more teeth removed. At the start of radiation treatment, significantly more current smokers (53%) had dental extraction compared with former smokers (28%) or never smokers (34%) (*p* = 0.003). Systemic therapy was used simultaneously with RT in 30 patients. The majority of patients were treated with IMRT (84.1%), and only 7 patients received 3D-CRT.

D_mean_ to the lower jaw was 40.5 Gy (range 21–62 Gy), D_max_ was 59 Gy (49–75 Gy), and the mean PTV dimension was 804 ccm (range 68–4838 ccm), respectively.

### Group 2

The median patient age at the time of RT was 71 years (range 42–88 years); 37 patients (82.2%) were male and 8 (17.8%) were female. Pre-RT dental treatment before radiotherapy was performed in 12 patients (26.7%). Systemic therapy was used simultaneously with RT in 29 patients. The majority of patients were treated with IMRT (95.6%), and only 1 patient received 3D-CRT.

D_mean_ to the lower jaw was 26.0 Gy (range 12.0–67.0 Gy), D_max_ was 56.0 Gy (32.0–77.0 Gy), and the mean PTV dimension was 796 ccm (range 170–1389 ccm), respectively.

All tested dosimetric variables were mutually related and associated with the risk of ORN. Patients with ORN had a significantly higher D_mean_, D_max_ and PTV than the control group: 40.5 Gy vs. 26.0 Gy (*p* = 0.043), 59.0 Gy vs. 56.0 Gy (*p* = 0.033) and 804 ccm vs. 796 ccm (*p* = 0.028). Statistically significant differences in univariate analyses were noted for D_mean_ > 45 Gy, D_max_ > 60 Gy, PTV touching > 40% lower jaw as well as the need for pre-RT dental therapy due to potential focus teeth. In the multivariate analysis pre-RT dental surgery (HR 4.5; 1.8–11.5) and dosimetric parameters including D_mean_ > 45 Gy (HR 2.4; 1.0–5.7), D_max_ > 60 Gy (HR 1.3; 1.1–2.8) and PTV > 40% touching lower jaw (HR 1.1; 1.0–1.1) were also significantly associated with ORN. Results of univariable and multivariable analysis are shown in Table 2.

**Table 2:** Univariable and multivariate analysis of risk factors for ORN

	HR	95% CI	*p*-value
**Univariate analysis**			
Gender (male vs. female)	1.837	0.847–2.984	0.124
Age	1.002	0.974–1.031	0.886
T-stage	1.320	0.962–1.813	0.086
Poor dental status (caries, periodontal disease)	1.618	0.740–3.537	0.228
Pre-RT dental treatment	4.822	2.055–11.316	**0.001**
Smoking history	1.279	0.672–2.435	0.453
D_mean_ to mandible	1.950	1.770–2.170	**0.043**
D_max_ to mandible	1.492	1.257–2.943	**0.033**
Concomitant systemic therapy	1.163	0.769–1.759	0.475
PTV dimension	1.001	1.000–1.003	**0.028**
**Multivariate analysis**			
Pre-RT dental treatment	4.567	1.817–11.477	**0.001**
D_mean_ to mandible	2.421	1.023–5.728	**0.044**
D_max_ to mandible	1.334	1.135–2.827	**0.018**
PTV dimension	1.001	1.000–1.003	**0.046**

## Discussion

ORN of the lower jaw is known to be a chronic late complication of RT in patients with oral cavity cancer, and many studies have reported its incidence. Incidences in several recent studies range between 4 and 8% [4–6, 15], and there has been a decrease over the past few decades since more conformal techniques (IMRT) have become available, as demonstrated by Studer et al. [15] and Ben-David et al. [10]. Also convincing are recent studies which clarify dosimetric prognostic factors for the incidence and severity of ORN.

Lee et al. and Niewald et al. reported on the dose–effect relationship in patients with oral cavity cancer. The frequency of ORN in their analyses was 6.6% [22] and 8.6% [23] in a median time of 2 years [22, 23]; in our analysis the follow-up duration was significantly lower for some patients. Higher radiation doses were significantly associated with the development of ORN [22, 23]. Lee et al. found that a cumulative total dose of > 54 Gy was a significant factor in the development of ORN [22, 24]. Nabil et al. reported that a total dose above 60 Gy was a significant parameter for ORN [12].

In our analysis, dosimetric parameters showed significant correlation with the development of ORN as seen with significant values in group 1: patients treated with D_max_ to lower jaw higher than 60 Gy (45.5% vs. 35.5%, *p* = 0.033), D_mean_ more than 45 Gy (43.25 vs. 8.9%, *p* = 0.023) and PTV intersection of more than 40% of the lower jaw bone. All patients with ORN had hot spots in the region of ORN. Dosimetric evaluation of D_mean_ in our study may reflect a general damaging mechanism in the lower jaw, and from this study, D_mean_ seems an appropriate parameter to consider in dose planning and be included without a threshold. Doses for the lower jaw bone should be kept as low as possible to reduce the risk of ORN in oral cavity cancer patients. Our general dose constraint (D_max_) for the lower jaw is 72 Gy or as low as achievable, although we do not compromise tumor coverage to achieve this value.

The median time to develop ORN in our study was 18 months. This is in line with other studies [6, 8, 12, 14, 25]. In a recent study of our institution there was a rate of ORN in 5.8% of patients detected [25]. An appropriate follow-up time was ensured in this retrospective study, and we include cases (group 1) and controls (group 2) for better correlation with factors leading to ORN in patients undergoing postoperative radiotherapy. Therefore, all patients had routine follow-up at our department as well as at the Department of Oral and Maxillofacial Surgery in Heidelberg. Our retrospective study showed that poor dental status which entails pre-RT dental treatment was significantly correlated with the development of ORN (70.5% vs. 26.7%). This is in line with previous studies [26].

The limitation of this study is its retrospective nature, which led to a shortage of necessary data on some single cases. However, we were able to retrieve follow-up data covering a lengthy time period for all patients. The power of this study is that we were able to show—in a dedicated sample of oral cavity cancer patients undergoing radiotherapy—risk factors for development of ORN: high risk was associated with poor dental status before beginning RT and with dosimetric parameters using D_max_ > 60 Gy, higher mean doses > 45 Gy and more than 40% of the PTV touching the lower jaw bone.

Further prospective studies are needed to further dissect the dental status and evaluate the infectivity of the focus teeth and the relationship of dental treatment and time interval with radiation onset. However, even if we assume that radiation contributes to the development of ORN at this dose constraint, there might be other contributory factors. The final goal would be to optimize radiation treatment plans with adaptive treatment planning and offer patients appropriate counseling for dental management to decrease the risk of ORN.

## Conclusion

The results of this retrospective study reveal that oral cavity cancer patients who underwent pre-RT dental surgery as well as dosimetric parameters using D_max_ > 60 Gy, Dmean > 45 Gy and more than 40% of the PTV touching the lower jaw bone are independent risk factors for the development of ORN. These findings can assist in the management of patients undergoing RT for head and neck cancer regarding ORN prevention. A larger study with focus on dental status is planned.

## Data Availability

Not applicable.
